# A case report of a huge congenital granular cell epulis operated under local anesthesia

**Published:** 2012-08-14

**Authors:** Khalid Khattala, Mohamed Rami, Aziz Elmadi, Leila Chbani, Youssef Bouabdallah

**Affiliations:** 1 Department of pediatric surgery, Hospital university Hassan II,Fez, Morroco; 2 Department of histopatghology, Hospital university Hassan II,Fez, Morroco

**Keywords:** Congenital granular cell tumor, gingival tumor, newborn, local anesthesia

## Abstract

Congenital granular cell epulis (CGCE) is a very rare benign soft tissue lesion of the neonate, it most frequently located on the anterior maxillary alveolar ridge. It has a female predilection. It is a tumor with no tendency to recur after excision. The exact histogenesis of this tumor remains unresolved and it may be hamartomata.

## Introduction

Congenital granular cell tumour (CGCT) was first described by Neumann in 1871 [1]. One hundred and sixty seven cases have been reported, with a maxillary to mandible ratio of 3:1 and a female to male ratio 10:1[2]. The etiology of the condition is not clear. Surgical excision is advocated as the treatment of choice for this tumor. We report a new case who presented an intraoral tumor mass which was protruding from her mouth, compromising feeding. The lesion was removed under local anesthesia with good follow-up.

## Patient and observation

**Figure 1:**
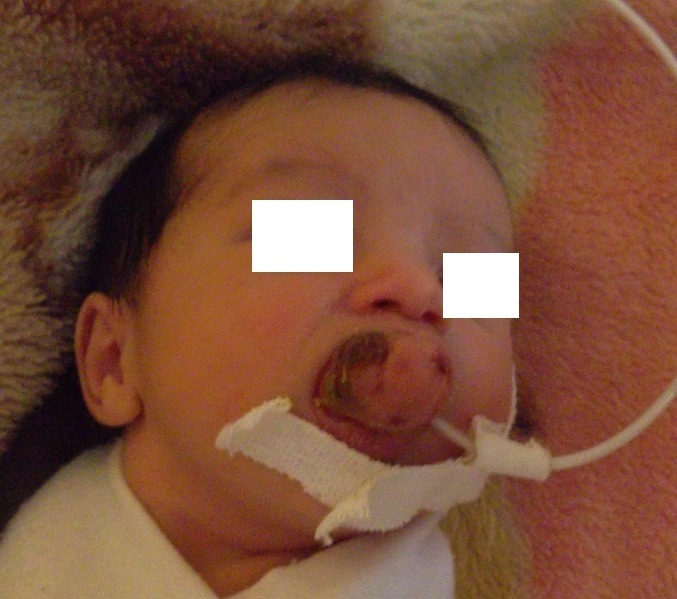
The preoperative appearance of the tumour showing It arising from the anterior maxillary alveolus (note the gastric tube for feeding)

A 2 day old girl, born at 41 weeks gestation weighting 3,500kg, was referred with a growth in the anterior maxillary alveolus which was noticed at birth. Clinical examination revealed a full term female neonate with a huge, exophytic growth protruding from the oral cavity, measured approximately 4x2 cm in size. The tumor was attached by a large pedicle to the anterior maxillary ridge; the mass was firm in consistency and not tender to digital palpation. The initial management included intravenous fluids and gastric feeding as oral feeding was impossible for this child (Figure 1).

**Figure 2:**
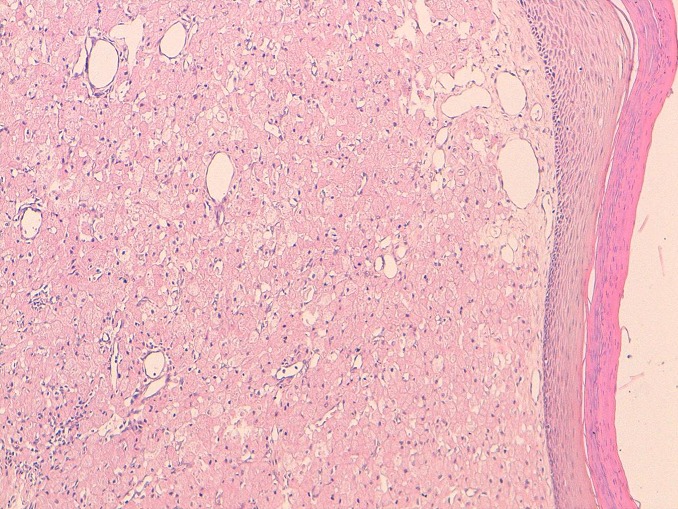
Section through the tumour (x 250) stained with haematoxylin and eosin, demonstrating characteristic polygonal cells with granular cytoplasm and small round nuclei

The tumor was excised under local anesthesia without a prior biopsy. No other lesions or systemic involvement was found. These were no complication after surgery. Histopathology confirmed the diagnosis of a completely excised congenital gingival granular cell tumour (Figure 2). Immunohistochemical investigation confirmed the diagnosis.

## Discussion

CGCT occurs in the mucosa of the maxillary alveolus as smooth surface pink mass, usually not associated with any other abnormality of the teeth or other congenital abnormalities [2]. It have usually been sporadically presented as isolated case reports in British literature, since the first case was described in Germany in 1871 as a congenital epulis by Neumann [1, 3]. The tumor is postulated to originate from indifferentiatedmesenchymal cell, fibroblasts, myofibroblasts, histiocyts, Schwann cells or odontogenic epithelial cells; it is a benign mesenchymal tumor of unknown origin [4]. CGCT is a very rare lesion that appears as a sessile or pedunculated lesions protruding from the neonate's mouth. The tumor occurs ten times more frequently in females than males and three times more frequently in the maxilla than mandible. It usually occurs as a single mass although 10% cases occur as multiple [5]. They are usually less than 1,5cm diameter [6]. Local excision is curative, with no reported recurrences in the literature, even when the excision has been incomplete [7], the CGCT removal under local anesthesia is an alternative if intubation is not possible us in our case or in case of small lesion [8]. Histopathology is the gold standard in the diagnostic process.

## Conclusion

CGCT is a very rare mouth tumor, the lesion can be removed under local anesthesia if intubation is not possible, and the diagnosis can be confirmed after histopathology study, without possibility of recurrence.
